# Retraction Note: Immune characteristics of children with autoimmune encephalitis and the correlation with a short-term prognosis

**DOI:** 10.1186/s13052-023-01566-w

**Published:** 2023-12-05

**Authors:** Jin-Yue Huang, Wen-Xuan Fan, Jing Meng, Chun-Quan Cai, Dong Li

**Affiliations:** 1 Tianjin Children’s Hospital, Tianjin University Children’s Hospital), Tianjin, 300134 China; 2 Department of Institute of Pediatrics, Tianjin Children’s Hospital, Tianjin, 300134 China; 3 Tianjin Key Laboratory of prevention and treatment of child birth defects, Tianjin, 300134 China; 4 Department of Neurology, Tianjin Children’s Hospital, No. 238 of Long-Yan Road, Bei-Chen District, Tianjin, 300134 China

**Retraction Note: Italian Journal of Pediatrics (2022) 48:94**

10.1186/s13052-022-01247-0

The Editor-in-Chief has retracted this article at the request of the corresponding author Dong Li because the authors have become aware of a number of errors in the data presented. Dong Li agrees with this retraction. Jin-Yue Huang, Wen-Xuan Fan, Jing Meng and Chun-Quan Cai have not responded to correspondence from the Publisher about this retraction.

